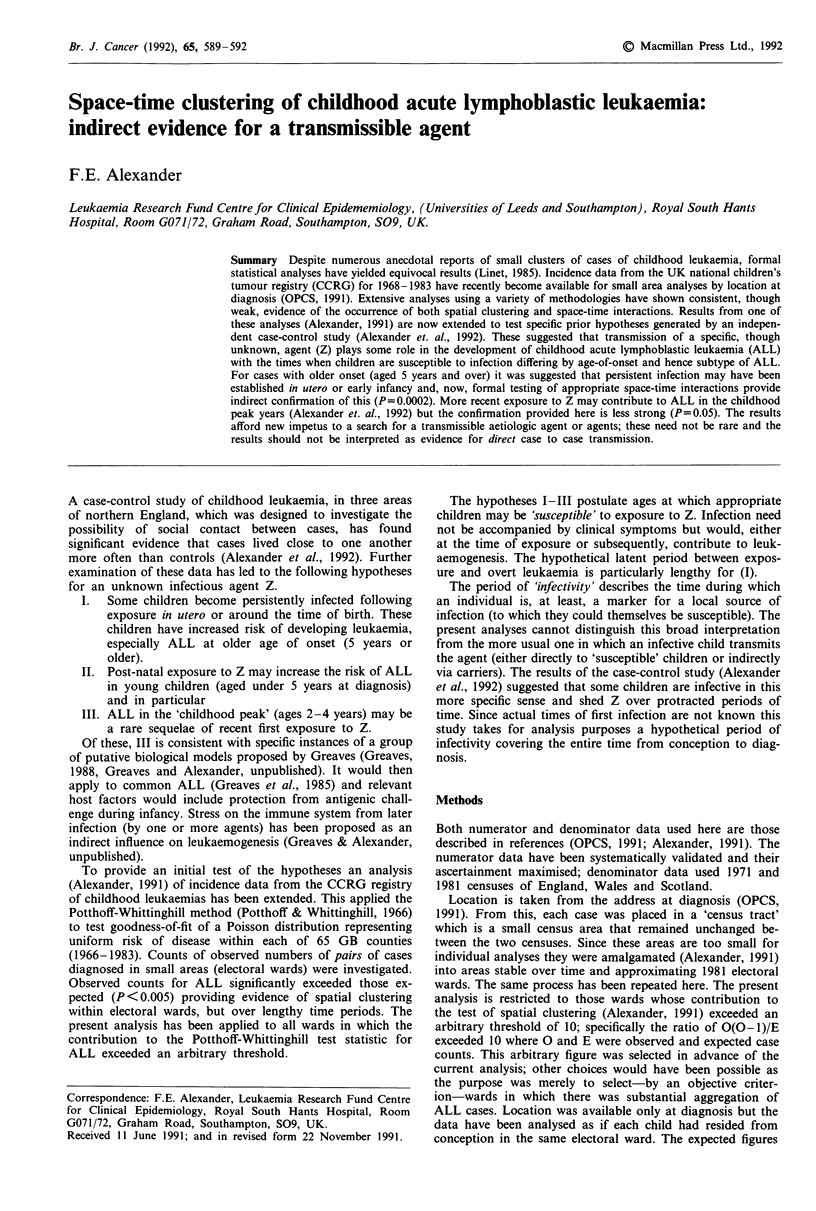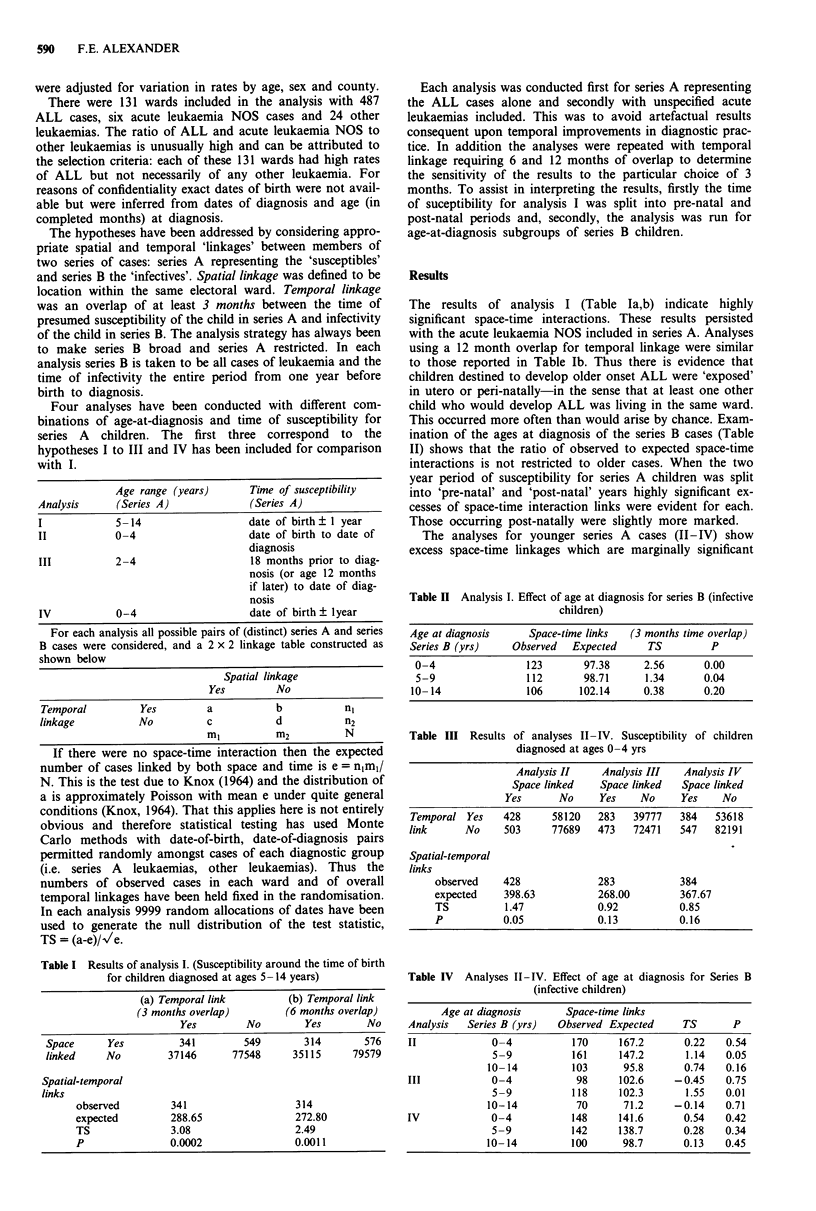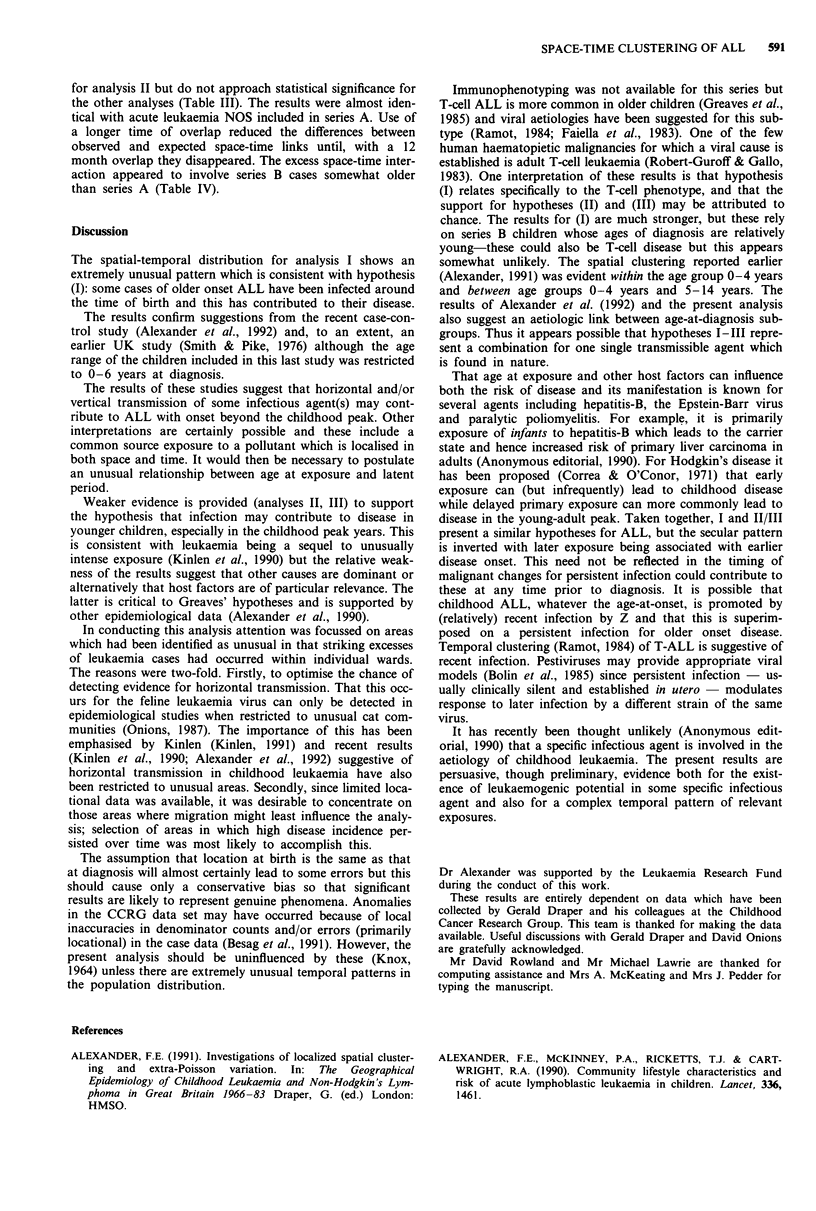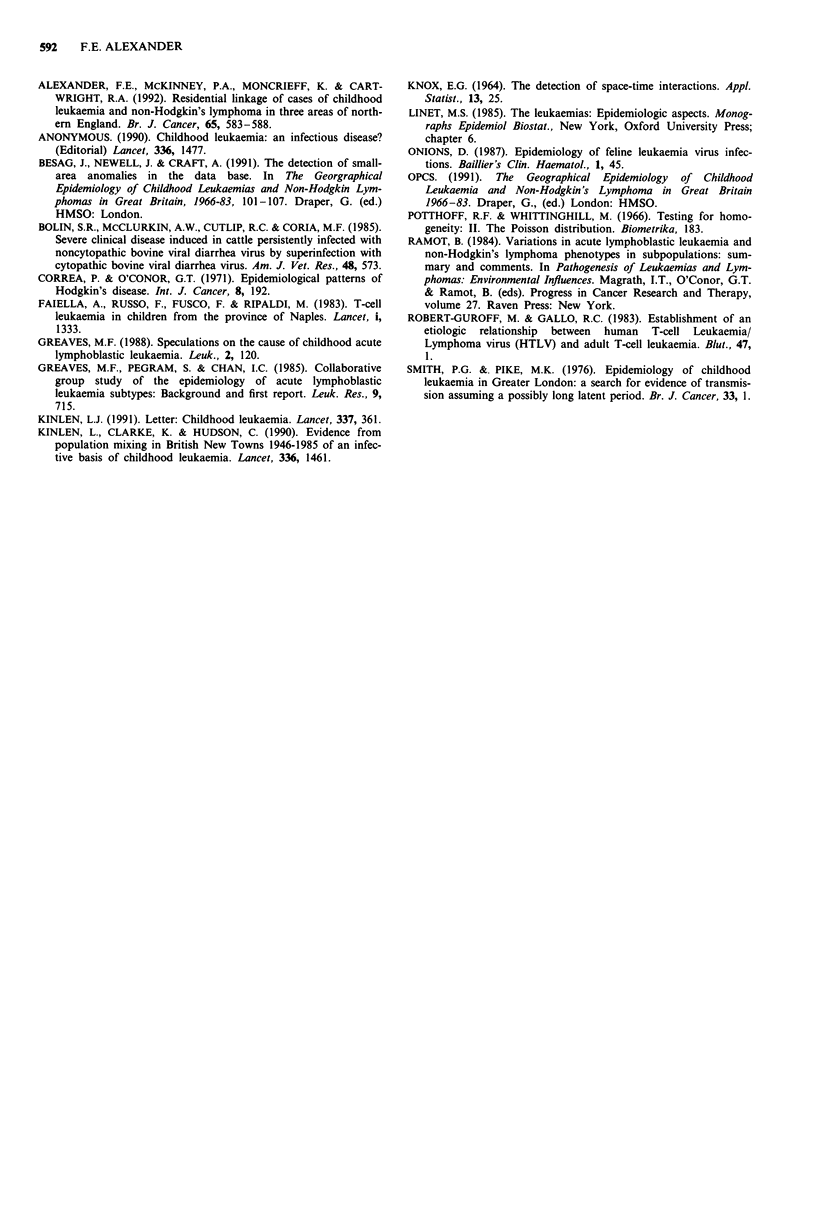# Space-time clustering of childhood acute lymphoblastic leukaemia: indirect evidence for a transmissible agent.

**DOI:** 10.1038/bjc.1992.119

**Published:** 1992-04

**Authors:** F. E. Alexander

**Affiliations:** Leukaemia Research Fund Centre for Clinical Epidemiology, University of Leeds, UK.

## Abstract

Despite numerous anecdotal reports of small clusters of cases of childhood leukaemia, formal statistical analyses have yielded equivocal results (Linet, 1985). Incidence data from the UK national children's tumour registry (CCRG) for 1968-1983 have recently become available for small area analyses by location at diagnosis (OPCS, 1991). Extensive analyses using a variety of methodologies have shown consistent, though weak, evidence of the occurrence of both spatial clustering and space-time interactions. Results from one of these analyses (Alexander, 1991) are now extended to test specific prior hypotheses generated by an independent case-control study (Alexander et. al., 1992). These suggested that transmission of a specific, though unknown, agent (Z) plays some role in the development of childhood acute lymphoblastic leukaemia (ALL) with the times when children are susceptible to infection differing by age-of-onset and hence subtype of ALL. For cases with older onset (aged 5 years and over) it was suggested that persistent infection may have been established in utero or early infancy and, now, formal testing of appropriate space-time interactions provide indirect confirmation of this (P = 0.0002). More recent exposure to Z may contribute to ALL in the childhood peak years (Alexander et. al., 1992) but the confirmation provided here is less strong (P = 0.05). The results afford new impetus to a search for a transmissible aetiologic agent or agents; these need not be rare and the results should not be interpreted as evidence for direct case to case transmission.


Br. J. Cancer (1992), 65, 589-592 © Macmillan Press Ltd., 1992

### Space-time clustering of childhood acute lymphoblastic leukaemia: indirect evidence for a transmissible agent

F.E. Alexander

*Leukaemia Research Fund Centre for Clinical Epidememiology, (Universities of Leeds and Southampton), Royal South Hants Hospital, Room G071/72, Graham Road, Southampton, SO9, UK.*

**Summary** Despite numerous anecdotal reports of small clusters of cases of childhood leukaemia, formal statistical analyses have yielded equivocal results (Linet, 1985). Incidence data from the UK national children's tumour registry (CCRG) for 1968-1983 have recently become available for small area analyses by location at diagnosis (OPCS, 1991). Extensive analyses using a variety of methodologies have shown consistent, though weak, evidence of the occurrence of both spatial clustering and space-time interactions. Results from one of these analyses (Alexander, 1991) are now extended to test specific prior hypotheses generated by an independent case-control study (Alexander *et. al.*, 1992). These suggested that transmission of a specific, though unknown, agent (Z) plays some role in the development of childhood acute lymphoblastic leukaemia (ALL) with the times when children are susceptible to infection differing by age-of-onset and hence subtype of ALL. For cases with older onset (aged 5 years and over) it was suggested that persistent infection may have been established *in utero* or early infancy and, now, formal testing of appropriate space-time interactions provide indirect confirmation of this ($P=0.0002$). More recent exposure to Z may contribute to ALL in the childhood peak years (Alexander *et. al.*, 1992) but the confirmation provided here is less strong ($P=0.05$). The results afford new impetus to a search for a transmissible aetiologic agent or agents; these need not be rare and the results should not be interpreted as evidence for *direct* case to case transmission.

A case-control study of childhood leukaemia, in three areas of northern England, which was designed to investigate the possibility of social contact between cases, has found significant evidence that cases lived close to one another more often than controls (Alexander *et al.*, 1992). Further examination of these data has led to the following hypotheses for an unknown infectious agent Z.

I. Some children become persistently infected following exposure *in utero* or around the time of birth. These children have increased risk of developing leukaemia, especially ALL at older age of onset (5 years or older).
II. Post-natal exposure to Z may increase the risk of ALL in young children (aged under 5 years at diagnosis) and in particular
III. ALL in the 'childhood peak' (ages 2-4 years) may be a rare sequelae of recent first exposure to Z.

Of these, III is consistent with specific instances of a group of putative biological models proposed by Greaves (Greaves, 1988, Greaves and Alexander, unpublished). It would then apply to common ALL (Greaves *et al.*, 1985) and relevant host factors would include protection from antigenic challenge during infancy. Stress on the immune system from later infection (by one or more agents) has been proposed as an indirect influence on leukaemogenesis (Greaves & Alexander, unpublished).

To provide an initial test of the hypotheses an analysis (Alexander, 1991) of incidence data from the CCRG registry of childhood leukaemias has been extended. This applied the Potthoff-Whittinghill method (Potthoff & Whittinghill, 1966) to test goodness-of-fit of a Poisson distribution representing uniform risk of disease within each of 65 GB counties (1966-1983). Counts of observed numbers of *pairs* of cases diagnosed in small areas (electoral wards) were investigated. Observed counts for ALL significantly exceeded those expected ($P<0.005$) providing evidence of spatial clustering within electoral wards, but over lengthy time periods. The present analysis has been applied to all wards in which the contribution to the Potthoff-Whittinghill test statistic for ALL exceeded an arbitrary threshold.

Correspondence: F.E. Alexander, Leukaemia Research Fund Centre for Clinical Epidemiology, Royal South Hants Hospital, Room G071/72, Graham Road, Southampton, SO9, UK.
Received 11 June 1991; and in revised form 22 November 1991.

The hypotheses I-III postulate ages at which appropriate children may be *'susceptible'* to exposure to Z. Infection need not be accompanied by clinical symptoms but would, either at the time of exposure or subsequently, contribute to leukaemogenesis. The hypothetical latent period between exposure and overt leukaemia is particularly lengthy for (I).

The period of *'infectivity'* describes the time during which an individual is, at least, a marker for a local source of infection (to which they could themselves be susceptible). The present analyses cannot distinguish this broad interpretation from the more usual one in which an infective child transmits the agent (either directly to 'susceptible' children or indirectly via carriers). The results of the case-control study (Alexander *et al.*, 1992) suggested that some children are infective in this more specific sense and shed Z over protracted periods of time. Since actual times of first infection are not known this study takes for analysis purposes a hypothetical period of infectivity covering the entire time from conception to diagnosis.

### Methods

Both numerator and denominator data used here are those described in references (OPCS, 1991; Alexander, 1991). The numerator data have been systematically validated and their ascertainment maximised; denominator data used 1971 and 1981 censuses of England, Wales and Scotland.

Location is taken from the address at diagnosis (OPCS, 1991). From this, each case was placed in a 'census tract' which is a small census area that remained unchanged between the two censuses. Since these areas are too small for individual analyses they were amalgamated (Alexander, 1991) into areas stable over time and approximating 1981 electoral wards. The same process has been repeated here. The present analysis is restricted to those wards whose contribution to the test of spatial clustering (Alexander, 1991) exceeded an arbitrary threshold of 10; specifically the ratio of O(O-1)/E exceeded 10 where O and E were observed and expected case counts. This arbitrary figure was selected in advance of the current analysis; other choices would have been possible as the purpose was merely to select—by an objective criterion—wards in which there was substantial aggregation of ALL cases. Location was available only at diagnosis but the data have been analysed as if each child had resided from conception in the same electoral ward. The expected figures were adjusted for variation in rates by age, sex and county.

There were 131 wards included in the analysis with 487 ALL cases, six acute leukaemia NOS cases and 24 other leukaemias. The ratio of ALL and acute leukaemia NOS to other leukaemias is unusually high and can be attributed to the selection criteria: each of these 131 wards had high rates of ALL but not necessarily of any other leukaemia. For reasons of confidentiality exact dates of birth were not available but were inferred from dates of diagnosis and age (in completed months) at diagnosis.

The hypotheses have been addressed by considering appropriate spatial and temporal 'linkages' between members of two series of cases: series A representing the 'susceptibles' and series B the 'infectives'. *Spatial linkage* was defined to be location within the same electoral ward. *Temporal linkage* was an overlap of at least *3 months* between the time of presumed susceptibility of the child in series A and infectivity of the child in series B. The analysis strategy has always been to make series B broad and series A restricted. In each analysis series B is taken to be all cases of leukaemia and the time of infectivity the entire period from one year before birth to diagnosis.

Four analyses have been conducted with different combinations of age-at-diagnosis and time of susceptibility for series A children. The first three correspond to the hypotheses I to III and IV has been included for comparison with I.

| *Analysis* | *Age range (years) (Series A)* | *Time of susceptibility (Series A)* |
|---|---|---|
| I | 5–14 | date of birth ± 1 year |
| II | 0–4 | date of birth to date of diagnosis |
| III | 2–4 | 18 months prior to diagnosis (or age 12 months if later) to date of diagnosis |
| IV | 0–4 | date of birth ± 1year |

For each analysis all possible pairs of (distinct) series A and series B cases were considered, and a 2 × 2 linkage table constructed as shown below

| | | *Spatial linkage* | | |
|---|---|---|---|---|
| | | *Yes* | *No* | |
| *Temporal* | *Yes* | a | b | $n_1$ |
| *linkage* | *No* | c | d | $n_2$ |
| | | $m_1$ | $m_2$ | N |

If there were no space-time interaction then the expected number of cases linked by both space and time is $e = n_1m_1/N$. This is the test due to Knox (1964) and the distribution of a is approximately Poisson with mean e under quite general conditions (Knox, 1964). That this applies here is not entirely obvious and therefore statistical testing has used Monte Carlo methods with date-of-birth, date-of-diagnosis pairs permitted randomly amongst cases of each diagnostic group (i.e. series A leukaemias, other leukaemias). Thus the numbers of observed cases in each ward and of overall temporal linkages have been held fixed in the randomisation. In each analysis 9999 random allocations of dates have been used to generate the null distribution of the test statistic, $TS = (a-e)/\sqrt{e}$.

**Table I** Results of analysis I. (Susceptibility around the time of birth for children diagnosed at ages 5–14 years)

| | | (a) *Temporal link (3 months overlap)* | | (b) *Temporal link (6 months overlap)* | |
|---|---|---|---|---|---|
| | | *Yes* | *No* | *Yes* | *No* |
| *Space* | *Yes* | 341 | 549 | 314 | 576 |
| *linked* | *No* | 37146 | 77548 | 35115 | 79579 |
| *Spatial-temporal links* | | | | | |
| observed | | 341 | | 314 | |
| expected | | 288.65 | | 272.80 | |
| TS | | 3.08 | | 2.49 | |
| *P* | | 0.0002 | | 0.0011 | |

Each analysis was conducted first for series A representing the ALL cases alone and secondly with unspecified acute leukaemias included. This was to avoid artefactual results consequent upon temporal improvements in diagnostic practice. In addition the analyses were repeated with temporal linkage requiring 6 and 12 months of overlap to determine the sensitivity of the results to the particular choice of 3 months. To assist in interpreting the results, firstly the time of suceptibility for analysis I was split into pre-natal and post-natal periods and, secondly, the analysis was run for age-at-diagnosis subgroups of series B children.

### Results

The results of analysis I (Table Ia,b) indicate highly significant space-time interactions. These results persisted with the acute leukaemia NOS included in series A. Analyses using a 12 month overlap for temporal linkage were similar to those reported in Table Ib. Thus there is evidence that children destined to develop older onset ALL were 'exposed' in utero or peri-natally—in the sense that at least one other child who would develop ALL was living in the same ward. This occurred more often than would arise by chance. Examination of the ages at diagnosis of the series B cases (Table II) shows that the ratio of observed to expected space-time interactions is not restricted to older cases. When the two year period of susceptibility for series A children was split into 'pre-natal' and 'post-natal' years highly significant excesses of space-time interaction links were evident for each. Those occurring post-natally were slightly more marked.

The analyses for younger series A cases (II–IV) show excess space-time linkages which are marginally significant

**Table II** Analysis I. Effect of age at diagnosis for series B (infective children)

| *Age at diagnosis Series B (yrs)* | *Space-time links Observed* | *Expected* | *(3 months time overlap) TS* | *P* |
|---|---|---|---|---|
| 0–4 | 123 | 97.38 | 2.56 | 0.00 |
| 5–9 | 112 | 98.71 | 1.34 | 0.04 |
| 10–14 | 106 | 102.14 | 0.38 | 0.20 |

**Table III** Results of analyses II–IV. Susceptibility of children diagnosed at ages 0–4 yrs

| | | *Analysis II Space linked* | | *Analysis III Space linked* | | *Analysis IV Space linked* | |
|---|---|---|---|---|---|---|---|
| | | *Yes* | *No* | *Yes* | *No* | *Yes* | *No* |
| *Temporal* | *Yes* | 428 | 58120 | 283 | 39777 | 384 | 53618 |
| *link* | *No* | 503 | 77689 | 473 | 72471 | 547 | 82191 |
| *Spatial-temporal links* | | | | | | | |
| observed | | 428 | | 283 | | 384 | |
| expected | | 398.63 | | 268.00 | | 367.67 | |
| TS | | 1.47 | | 0.92 | | 0.85 | |
| *P* | | 0.05 | | 0.13 | | 0.16 | |

**Table IV** Analyses II–IV. Effect of age at diagnosis for Series B (infective children)

| *Analysis* | *Age at diagnosis Series B (yrs)* | *Space-time links Observed* | *Expected* | *TS* | *P* |
|---|---|---|---|---|---|
| II | 0–4 | 170 | 167.2 | 0.22 | 0.54 |
| | 5–9 | 161 | 147.2 | 1.14 | 0.05 |
| | 10–14 | 103 | 95.8 | 0.74 | 0.16 |
| III | 0–4 | 98 | 102.6 | −0.45 | 0.75 |
| | 5–9 | 118 | 102.3 | 1.55 | 0.01 |
| | 10–14 | 70 | 71.2 | −0.14 | 0.71 |
| IV | 0–4 | 148 | 141.6 | 0.54 | 0.42 |
| | 5–9 | 142 | 138.7 | 0.28 | 0.34 |
| | 10–14 | 100 | 98.7 | 0.13 | 0.45 |

for analysis II but do not approach statistical significance for the other analyses (Table III). The results were almost identical with acute leukaemia NOS included in series A. Use of a longer time of overlap reduced the differences between observed and expected space-time links until, with a 12 month overlap they disappeared. The excess space-time interaction appeared to involve series B cases somewhat older than series A (Table IV).

### Discussion

The spatial-temporal distribution for analysis I shows an extremely unusual pattern which is consistent with hypothesis (I): some cases of older onset ALL have been infected around the time of birth and this has contributed to their disease.

The results confirm suggestions from the recent case-control study (Alexander *et al.*, 1992) and, to an extent, an earlier UK study (Smith & Pike, 1976) although the age range of the children included in this last study was restricted to 0–6 years at diagnosis.

The results of these studies suggest that horizontal and/or vertical transmission of some infectious agent(s) may contribute to ALL with onset beyond the childhood peak. Other interpretations are certainly possible and these include a common source exposure to a pollutant which is localised in both space and time. It would then be necessary to postulate an unusual relationship between age at exposure and latent period.

Weaker evidence is provided (analyses II, III) to support the hypothesis that infection may contribute to disease in younger children, especially in the childhood peak years. This is consistent with leukaemia being a sequel to unusually intense exposure (Kinlen *et al.*, 1990) but the relative weakness of the results suggest that other causes are dominant or alternatively that host factors are of particular relevance. The latter is critical to Greaves' hypotheses and is supported by other epidemiological data (Alexander *et al.*, 1990).

In conducting this analysis attention was focussed on areas which had been identified as unusual in that striking excesses of leukaemia cases had occurred within individual wards. The reasons were two-fold. Firstly, to optimise the chance of detecting evidence for horizontal transmission. That this occurs for the feline leukaemia virus can only be detected in epidemiological studies when restricted to unusual cat communities (Onions, 1987). The importance of this has been emphasised by Kinlen (Kinlen, 1991) and recent results (Kinlen *et al.*, 1990; Alexander *et al.*, 1992) suggestive of horizontal transmission in childhood leukaemia have also been restricted to unusual areas. Secondly, since limited locational data was available, it was desirable to concentrate on those areas where migration might least influence the analysis; selection of areas in which high disease incidence persisted over time was most likely to accomplish this.

The assumption that location at birth is the same as that at diagnosis will almost certainly lead to some errors but this should cause only a conservative bias so that significant results are likely to represent genuine phenomena. Anomalies in the CCRG data set may have occurred because of local inaccuracies in denominator counts and/or errors (primarily locational) in the case data (Besag *et al.*, 1991). However, the present analysis should be uninfluenced by these (Knox, 1964) unless there are extremely unusual temporal patterns in the population distribution.

Immunophenotyping was not available for this series but T-cell ALL is more common in older children (Greaves *et al.*, 1985) and viral aetiologies have been suggested for this subtype (Ramot, 1984; Faiella *et al.*, 1983). One of the few human haematopietic malignancies for which a viral cause is established is adult T-cell leukaemia (Robert-Guroff & Gallo, 1983). One interpretation of these results is that hypothesis (I) relates specifically to the T-cell phenotype, and that the support for hypotheses (II) and (III) may be attributed to chance. The results for (I) are much stronger, but these rely on series B children whose ages of diagnosis are relatively young—these could also be T-cell disease but this appears somewhat unlikely. The spatial clustering reported earlier (Alexander, 1991) was evident *within* the age group 0–4 years and *between* age groups 0–4 years and 5–14 years. The results of Alexander *et al.* (1992) and the present analysis also suggest an aetiologic link between age-at-diagnosis subgroups. Thus it appears possible that hypotheses I–III represent a combination for one single transmissible agent which is found in nature.

That age at exposure and other host factors can influence both the risk of disease and its manifestation is known for several agents including hepatitis-B, the Epstein-Barr virus and paralytic poliomyelitis. For example, it is primarily exposure of *infants* to hepatitis-B which leads to the carrier state and hence increased risk of primary liver carcinoma in adults (Anonymous editorial, 1990). For Hodgkin's disease it has been proposed (Correa & O'Conor, 1971) that early exposure can (but infrequently) lead to childhood disease while delayed primary exposure can more commonly lead to disease in the young-adult peak. Taken together, I and II/III present a similar hypotheses for ALL, but the secular pattern is inverted with later exposure being associated with earlier disease onset. This need not be reflected in the timing of malignant changes for persistent infection could contribute to these at any time prior to diagnosis. It is possible that childhood ALL, whatever the age-at-onset, is promoted by (relatively) recent infection by Z and that this is superimposed on a persistent infection for older onset disease. Temporal clustering (Ramot, 1984) of T-ALL is suggestive of recent infection. Pestiviruses may provide appropriate viral models (Bolin *et al.*, 1985) since persistent infection — usually clinically silent and established *in utero* — modulates response to later infection by a different strain of the same virus.

It has recently been thought unlikely (Anonymous editorial, 1990) that a specific infectious agent is involved in the aetiology of childhood leukaemia. The present results are persuasive, though preliminary, evidence both for the existence of leukaemogenic potential in some specific infectious agent and also for a complex temporal pattern of relevant exposures.

Dr Alexander was supported by the Leukaemia Research Fund during the conduct of this work.

These results are entirely dependent on data which have been collected by Gerald Draper and his colleagues at the Childhood Cancer Research Group. This team is thanked for making the data available. Useful discussions with Gerald Draper and David Onions are gratefully acknowledged.

Mr David Rowland and Mr Michael Lawrie are thanked for computing assistance and Mrs A. McKeating and Mrs J. Pedder for typing the manuscript.

### References

ALEXANDER, F.E. (1991). Investigations of localized spatial clustering and extra-Poisson variation. In: *The Geographical Epidemiology of Childhood Leukaemia and Non-Hodgkin's Lymphoma in Great Britain 1966–83* Draper, G. (ed.) London: HMSO.

ALEXANDER, F.E., MCKINNEY, P.A., RICKETTS, T.J. & CARTWRIGHT, R.A. (1990). Community lifestyle characteristics and risk of acute lymphoblastic leukaemia in children. *Lancet*, **336**, 1461.

ALEXANDER, F.E., MCKINNEY, P.A., MONCRIEFF, K. & CARTWRIGHT, R.A. (1992). Residential linkage of cases of childhood leukaemia and non-Hodgkin's lymphoma in three areas of northern England. *Br. J. Cancer*, **65**, 583–588.

ANONYMOUS. (1990). Childhood leukaemia: an infectious disease? (Editorial) *Lancet*, **336**, 1477.

BESAG, J., NEWELL, J. & CRAFT, A. (1991). The detection of small-area anomalies in the data base. In *The Georgraphical Epidemiology of Childhood Leukaemias and Non-Hodgkin Lymphomas in Great Britain, 1966-83*, 101–107. Draper, G. (ed.) HMSO: London.

BOLIN, S.R., MCCLURKIN, A.W., CUTLIP, R.C. & CORIA, M.F. (1985). Severe clinical disease induced in cattle persistently infected with noncytopathic bovine viral diarrhea virus by superinfection with cytopathic bovine viral diarrhea virus. *Am. J. Vet. Res.*, **48**, 573.

CORREA, P. & O'CONOR, G.T. (1971). Epidemiological patterns of Hodgkin's disease. *Int. J. Cancer*, **8**, 192.

FAIELLA, A., RUSSO, F., FUSCO, F. & RIPALDI, M. (1983). T-cell leukaemia in children from the province of Naples. *Lancet*, **i**, 1333.

GREAVES, M.F. (1988). Speculations on the cause of childhood acute lymphoblastic leukaemia. *Leuk.*, **2**, 120.

GREAVES, M.F., PEGRAM, S. & CHAN, I.C. (1985). Collaborative group study of the epidemiology of acute lymphoblastic leukaemia subtypes: Background and first report. *Leuk. Res.*, **9**, 715.

KINLEN, L.J. (1991). Letter: Childhood leukaemia. *Lancet*, **337**, 361.

KINLEN, L., CLARKE, K. & HUDSON, C. (1990). Evidence from population mixing in British New Towns 1946-1985 of an infective basis of childhood leukaemia. *Lancet*, **336**, 1461.

KNOX, E.G. (1964). The detection of space-time interactions. *Appl. Statist.*, **13**, 25.

LINET, M.S. (1985). The leukaemias: Epidemiologic aspects. *Monographs Epidemiol Biostat.*, New York, Oxford University Press; chapter 6.

ONIONS, D. (1987). Epidemiology of feline leukaemia virus infections. *Baillier's Clin. Haematol.*, **1**, 45.

OPCS. (1991). *The Geographical Epidemiology of Childhood Leukaemia and Non-Hodgkin's Lymphoma in Great Britain 1966–83*. Draper, G., (ed.) London: HMSO.

POTTHOFF, R.F. & WHITTINGHILL, M. (1966). Testing for homogeneity: II. The Poisson distribution. *Biometrika*, 183.

RAMOT, B. (1984). Variations in acute lymphoblastic leukaemia and non-Hodgkin's lymphoma phenotypes in subpopulations: summary and comments. In *Pathogenesis of Leukaemias and Lymphomas: Environmental Influences*. Magrath, I.T., O'Conor, G.T. & Ramot, B. (eds). Progress in Cancer Research and Therapy, volume 27. Raven Press: New York.

ROBERT-GUROFF, M. & GALLO, R.C. (1983). Establishment of an etiologic relationship between human T-cell Leukaemia/Lymphoma virus (HTLV) and adult T-cell leukaemia. *Blut.*, **47**, 1.

SMITH, P.G. &. PIKE, M.K. (1976). Epidemiology of childhood leukaemia in Greater London: a search for evidence of transmission assuming a possibly long latent period. *Br. J. Cancer*, **33**, 1.